# Stable multi-GeV electron accelerator driven by waveform-controlled PW laser pulses

**DOI:** 10.1038/s41598-017-09267-1

**Published:** 2017-08-31

**Authors:** Hyung Taek Kim, V. B. Pathak, Ki Hong Pae, A. Lifschitz, F. Sylla, Jung Hun Shin, C. Hojbota, Seong Ku Lee, Jae Hee Sung, Hwang Woon Lee, E. Guillaume, C. Thaury, Kazuhisa Nakajima, J. Vieira, L. O. Silva, V. Malka, Chang Hee Nam

**Affiliations:** 1 Center for Relativistic Laser Science, Institute for Basic Science (IBS), Gwangju, 61005 Korea; 2 Advanced Photonics Research Institute, GIST, Gwangju, 61005 Korea; 3 Laboratoire d’Optique Appliquée (LOA), ENSTA ParisTech, CNRS UMR7639, École Polytechnique, Université Paris-Saclay, 828 Boulevard des Maréchaux, 91762 Palaiseau, France; 4 SourceLAB SAS, 86 rue de Paris, 91400 Orsay, France; 5 GoLP/Instituto de Plasmas e Fusão Nuclear, Instituto Superior Técnico, Universidade de Lisboa, Lisbon, Portugal; 6 Weizmann Institue for Science, P.O. Box 26, Rehovot, 76100 Israel; 7 Departement of Physics and Photon Science, GIST, Gwangju, 61005 Korea

## Abstract

The achievable energy and the stability of accelerated electron beams have been the most critical issues in laser wakefield acceleration. As laser propagation, plasma wave formation and electron acceleration are highly nonlinear processes, the laser wakefield acceleration (LWFA) is extremely sensitive to initial experimental conditions. We propose a simple and elegant waveform control method for the LWFA process to enhance the performance of a laser electron accelerator by applying a fully optical and programmable technique to control the chirp of PW laser pulses. We found sensitive dependence of energy and stability of electron beams on the spectral phase of laser pulses and obtained stable 2-GeV electron beams from a 1-cm gas cell of helium. The waveform control technique for LWFA would prompt practical applications of centimeter-scale GeV-electron accelerators to a compact radiation sources in the x-ray and γ-ray regions.

## Introduction

Laser wakefield electron accelerators^1^ have strong potential for a next generation accelerator due to its huge acceleration field. Laser wakefield acceleration (LWFA) has been intensively investigated to produce quasi-mono-energetic collimated GeV electron beams in centimeter-scale acceleration length^2–4^. Recent progresses of LWFA demonstrated quasi-mono-energetic electron beams in the hundreds MeV range that are produced in a stable way by utilizing methods such as colliding laser pulses^5, 6^, ionization injection^7–10^ and density gradient injection^11–14^ with an improvement of the stability that results from explored injection schemes. Here we propose a simple method to enhance the energy and the stability of multi-GeV electron beams^15–17^ by controlling the waveform of PW laser pulses using a fully optical, programmable method^18–20^.

LWFA is based on the interaction between an underdense plasma and an intense laser pulse. The interaction between a driving laser pulse and a plasma in LWFA is a complex process occurring in the relativistic regime, because the formation of plasma waves and electron acceleration processes are highly nonlinear while the propagating laser pulse is strongly modified by the medium^21, 22^. The LWFA is, thus, very sensitive to the initial properties of driving laser pulse, and the waveform of the laser pulse can significantly affect the LWFA process. The waveform control technique brought significant advancement of light-matter interactions by realizing coherent control^23, 24^ of a chemical reaction or atomic excitation/ionization. In the coherent control of a quantum system, the probability of a certain reaction or excitation/ionization pathway can be selectively enhanced by controlling the waveform of a driving laser pulse. Here, we propose and demonstrate that the LWFA can be optimized by controlling the waveform of the driving laser pulse so as to enhance the energy and the stability of accelerated electron beams; the laser-plasma interaction in the LWFA is strongly affected by the coherent property of the laser pulse.

## Results

### Waveform control of PW laser pulses

The waveform of PW laser pulse for LWFA was controlled by adopting an acousto-optic device. Previous studies on the effects of laser chirp^25–28^ were performed by mechanically detuning the grating pair of a pulse compressor. In this grating adjustment scheme there is a difficulty in distinguishing the effects of different spectral phase components, since the spectral phase terms cannot be individually adjusted. In our approach, we controlled independently each component of spectral phase terms in order to clearly observe the effect of laser chirp on the LWFA process.

The temporal structure of a laser pulse can be manipulated by individually changing spectral phase terms^29^. The spectral phase, φ(ω), can be expressed in terms of a Taylor series around the center frequency ${{\rm{\omega }}}_{0}$ of a laser spectrum. The group delay dispersion (GDD), ∂^2^ϕ(ω_0_)/∂ω^2^, is the spectral phase term describing linear chirp. The GDD makes symmetric temporal broadening; in the case of a positive GDD, i.e. a positively chirped pulse, the laser pulse has linearly increasing frequency composition in time, and vice versa for a negative GDD. The next order term, ∂^3^ϕ(ω_0_)/∂ω^3^, is denoted as the third order dispersion (TOD), or equivalently quadratic chirp, which produces an asymmetric temporal profile. By synthesizing spectral phase terms, we can adjust the instantaneous frequency composition and, subsequently, the electric field profile of a PW laser pulse.

**Figure 1:**
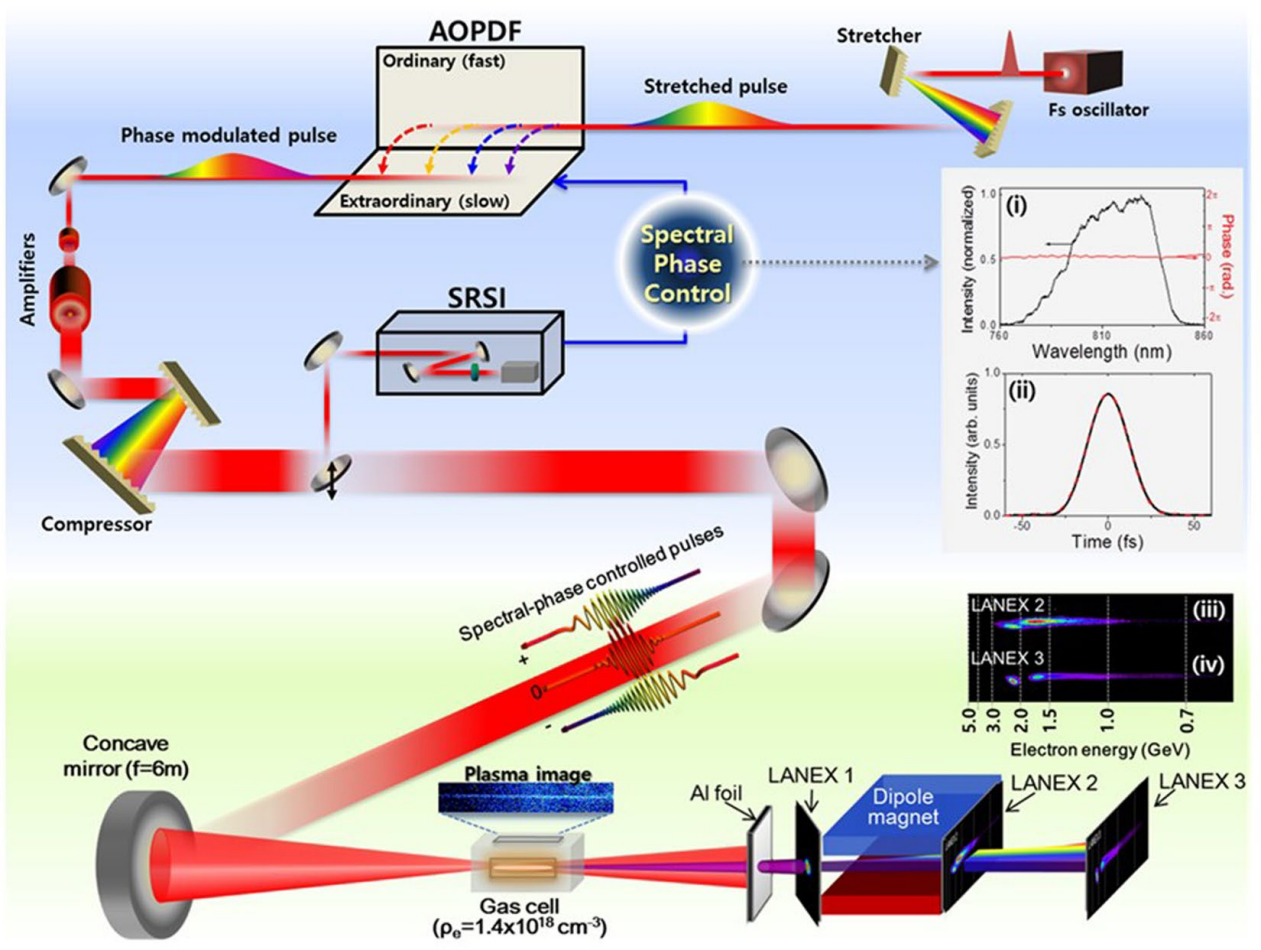
Schematic layout of LWFA experiments performed with the spectral phase control of PW laser pulses. The insets (i) and (ii) show the laser spectrum with flattened spectral phase and the temporal profile, respectively, after the feedback compensation of the spectral phase using SRSI and AOPDF. In (i) the measured spectral intensity and the phase are shown in the black and the red lines, respectively. In (ii) the temporal profile from measured spectral phase, shown in the black line, is hardly distinguishable from the transform-limited case shown in the red. The insets (iii) and (iv) show typical electron spectra measured at two positions- one at the end of the magnet and the other at 0.53 meter away.

In order to control the LWFA process, we applied a fully optical and programmable method^18–20^ to adjust the spectral phase of a PW laser pulse. This method allows the adjustment of a specific component of the spectral phase, distinguished from the grating detuning method. We, first, produced a flat spectral phase using an iterative feedback of the spectral phase, precisely measured with a self-referenced spectral interferometer (SRSI)^19^ installed after the pulse compressor of the PW laser, to an acousto-optic programmable dispersive filter (AOPDF)^18^ that can control the individual component of spectral phase terms, as shown in Fig. 1. We, then, set a specific spectral phase of the laser pulse using the AOPDF. The details of the PW laser and the experimental setup are described in Methods. We investigated the effect of the driving laser waveform on LWFA by actively controlling spectral phase components.

### Enhancement of LWFA by waveform control

**Figure 2:**
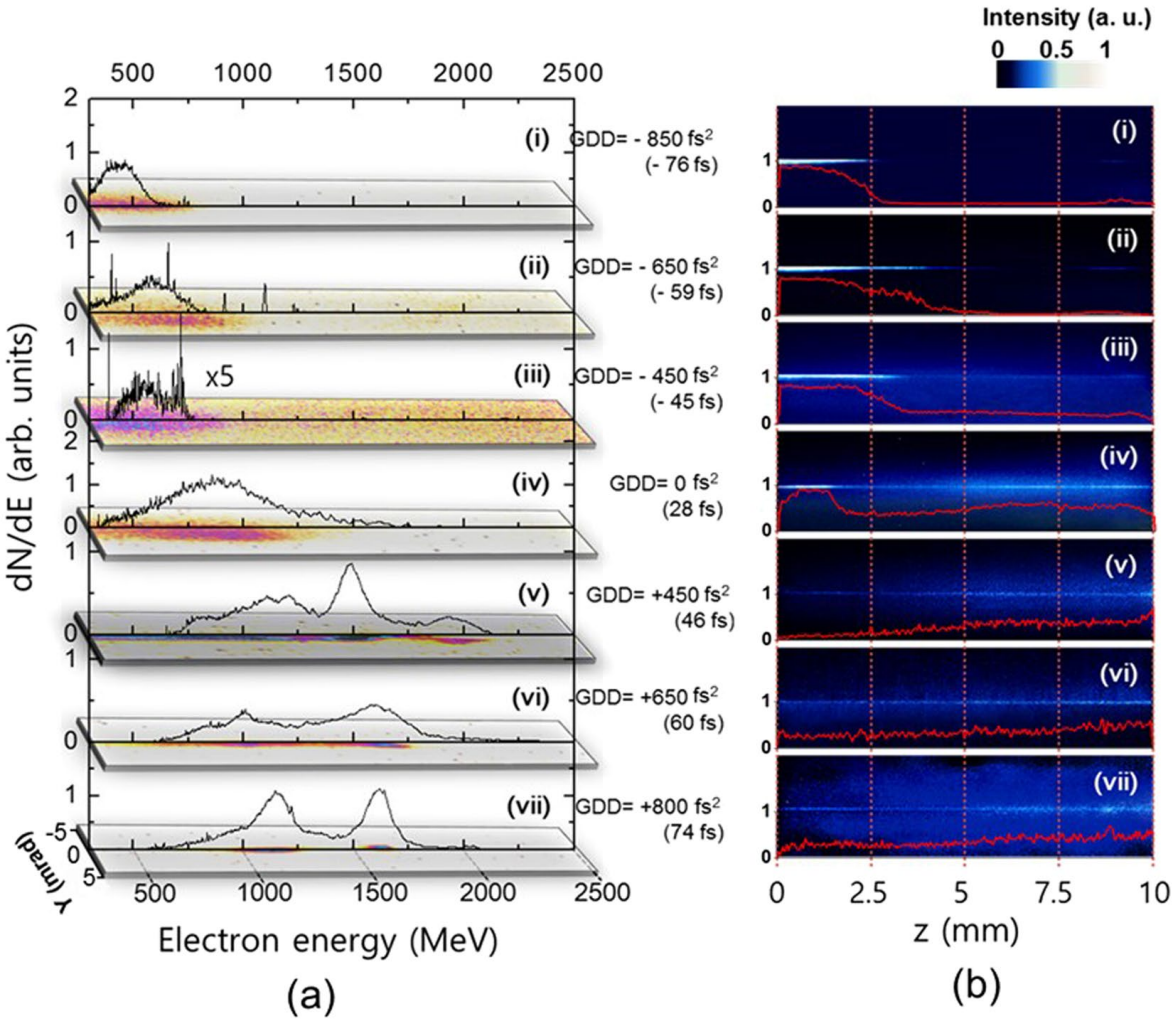
(**a**) Electron energy spectra obtained with different GDD’s of PW laser pulses. The GDD values are noted in the graph and pulse durations in FWHM for the GDD values are indicated in parentheses. The bottom image in the graph shows the spectral image of the electron beam for each GDD value. The length of the plasma medium is 10 mm. (**b**) Thomson scattering images measured at the orthogonal direction to the laser polarization and the propagation directions for different GDD’s. The red plots are the line profile of Thomson scattering signal along the laser propagation.

We systematically investigated the influence of laser chirp on LWFA^30^ in the bubble regime by employing the programmable feedback system for controlling the spectral phase. As the first step, we controlled GDD of the laser pulse and found that the electron energy sensitively changed with GDD, as shown in Fig. 2a. The length of the plasma medium in Fig. 2 is 10 mm. In the chirp-free case, the electron spectrum shows an energy peak near 1 GeV with broad energy spread, and the peak energies were significantly reduced to 0.5 GeV with negative GDD’s. As the laser chirp was controlled to positive GDD’s, the electron energy increased drastically to 1.9 GeV at the highest-energy peak with the positive GDD value of 450 fs^2^. Consequently, the experimental results clearly showed the sensitive dependence of electron energy on the laser chirp, signifying that the LWFA process can be controlled by applying chirped PW laser pulses^30–32^.

The propagation of chirped laser pulses through the plasma generated in the He gas cell was characterized by observing the Thomson scattering signal of propagating laser pulses^33^. The Thomson scattering images in Fig. 2b show the formation of plasma channels depending on laser chirp. The images from positively chirped pulses were narrow and uniform throughout the entire 10-mm medium, while those from negatively chirped pulses showed a strong signal in the first part of propagation and diminished suddenly. This observation indicates that positively chirped pulses are much preferable for a long and stable plasma channel formation throughout the medium, which is essential for the efficient acceleration of electrons.

**Figure 3:**
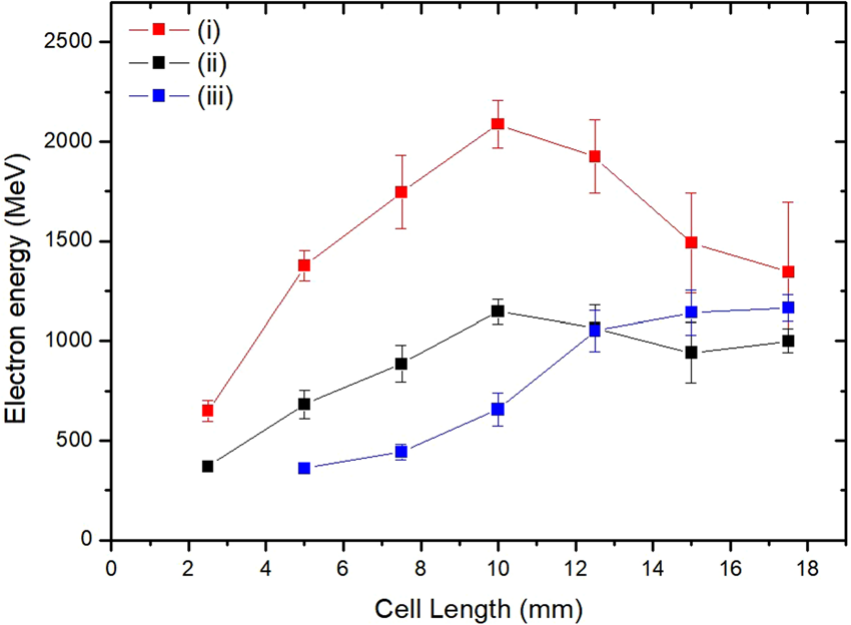
Variation of the highest peak electron energy obtained while changing the gas cell length for the cases of (i) positively chirped 40-fs, (ii) chirp-free 28-fs, and (iii) negatively chirped 40-fs laser pulses. The error bars indicate the standard deviation of peak electron energy variation of 10 shots. Error bars for the data points below 400 MeV near the minimum observable energy is not presented.

The electron energy variation for three different laser chirps - positively chirped 40 fs, chirp-free 28 fs, and negatively chirped 40 fs laser pulses - are reported in Fig. 3, as a function of the medium length. In the case of the positively chirped pulse, the electron energy increased rapidly up to the medium length of 10 mm, giving the acceleration gradient of about 2 GeV/cm, and decreased when the medium length increased further due to dephasing. In contrast, the acceleration gradient for the negatively chirped pulse was about 0.7 GeV/cm and the electron energy slowly increased up to 1 GeV for the medium length of 18 mm. For the chirp-free case, the gradient was about 1 GeV/cm up to 10-mm length and then the energy gain was almost saturated afterwards. These results show that the acceleration gradient depends sensitively on laser chirp; the positively chirped pulse generated stronger acceleration gradient in the acceleration phase than the chirp-free or negatively chirped pulse. In the nonlinear regime of LWFA, the leading edge of the laser pulse plays a dominant role in the creation of a plasma bubble. In the case of the positively chirped pulse electrons are strongly pushed by a strong ponderomotive force due to the long wavelength component in the leading edge, providing well-defined electron injection and high acceleration gradient during the early stage of acceleration. From the two-dimensional particle-in-cell simulations performed with OSIRIS^34^, as described in Supplementary, we obtained also a stronger and more stable bubble structure in the case of the positively chirped pulse, preferable conditions for electron acceleration, than in the case of the negatively chirped pulse.

**Figure 4:**
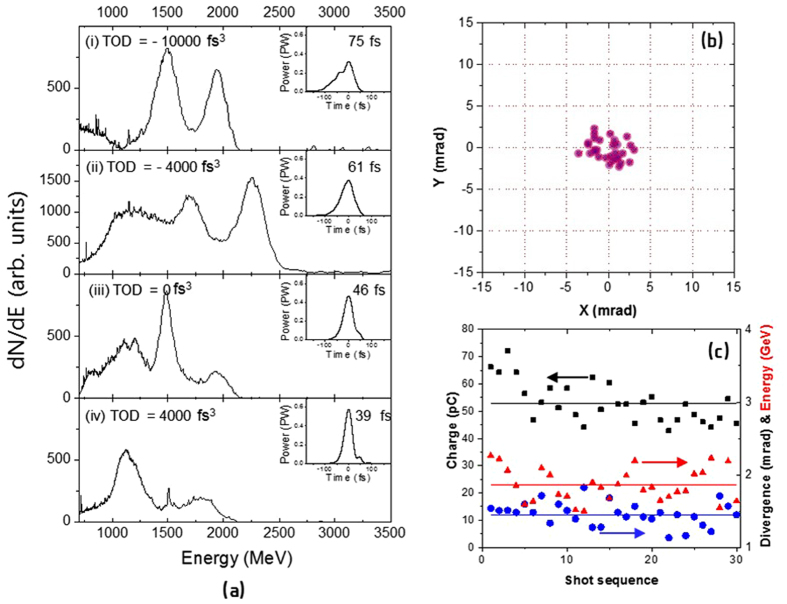
(**a**) Electron energy spectra obtained with different TOD’s for the positively chirped laser pulses with GDD of +450 fs^2^. The TOD values are given in the figure. The inset graph contains the temporal profile and pulse duration in FWHM of the laser pulse for given TOD. (**b**) Beam pointing and (**c**) total charge, divergence and peak energy of electron beams taken from 30 consecutive laser shots with GDD of +450 fs^2^ and TOD of −4000 fs^3^. The black, red and blue symbols are the total charge, peak energy and divergence, respectively. The total charge was monitored by the total CCD count of the electron beam profile. The individual symbol in (**c**) indicates the value for each shot and the lines show the averaged values.

For further control of the LWFA, we adjusted the quadratic chirp, i.e. TOD, while keeping the GDD value, 450 fs^2^, corresponding to the highest energy in Fig. 2a. Figure 4 shows the electron energy spectra and the laser temporal profiles obtained with different TOD values. The results show that the electron energy could be enhanced further by applying negative TOD’s, while the electron energy decreased in the case of positive TOD’s. The best spectral phase condition for highest electron energy was obtained with the TOD value of −4000 fs^3^. At this optimal spectral phase with the positive GDD and negative TOD, the temporal profile of the laser pulse had a slow-rising and fast-falling shape; an electron beam was obtained with the highest peak energy at 2.3 GeV with an energy spread of about 10%. The charge of the electron beam^35^ was about 70 pC for the electron energy over 650 MeV. The experimental result shows that, in addition to the GDD control, the manipulation of TOD could enhance the electron energy further.

We examined the stability of electron beams generated at the spectral phase condition that maximizes the electron energy. The energy spectra and spatial profiles of the electron beams were measured for 30 consecutive shots. As shown in Fig. 4(b and c), all the measured parameters of energy, total charge and divergence were stabilized at this optimized spectral phase condition. The pointing stabilities in the horizontal and the vertical directions were only 1.7 mrad and 1.2 mrad in standard deviation (SD), respectively. The average beam divergence was 1.4 mrad in full-width at half-maximum (FWHM) with 16% variation. The average energy of electron beams was about 1.9 GeV with an energy fluctuation of 12% in SD. In addition, the total charge of the electron beam had a fluctuation level of 14% in SD. The pointing fluctuation was very comparable to the beam divergence, indicating that the electron beam was stably directed to a certain point. The stable beam pointing is of practical importance in utilizing electron beams for applications. In the opposite case of a negatively chirped fast-rising pulse (pulse duration = 59 fs, GDD = −400 fs^2^, TOD = + 7000 fs^3^), we observed in the experiment that the electron beam was quite unstable, having peak energy about 600 MeV with 30-% variation, the pointing stability of 6 mrad, and the total charge fluctuation of 40%. For the chirp-free case, the electron beam with energy about 1 GeV has energy deviation of 15% and total charge fluctuation of 35% as well as pointing fluctuation of 3.5 mrad in SD. Consequently, we could enhance the energy, the beam pointing and the total charge of the accelerated electron beam by adjusting GDD and TOD of PW laser pulses; the LWFA process was optimized through the waveform control of driving laser pulses to achieve multi-GeV electron beams with excellent stability in energy, total charge and spatial beam quality.

**Figure 5:**
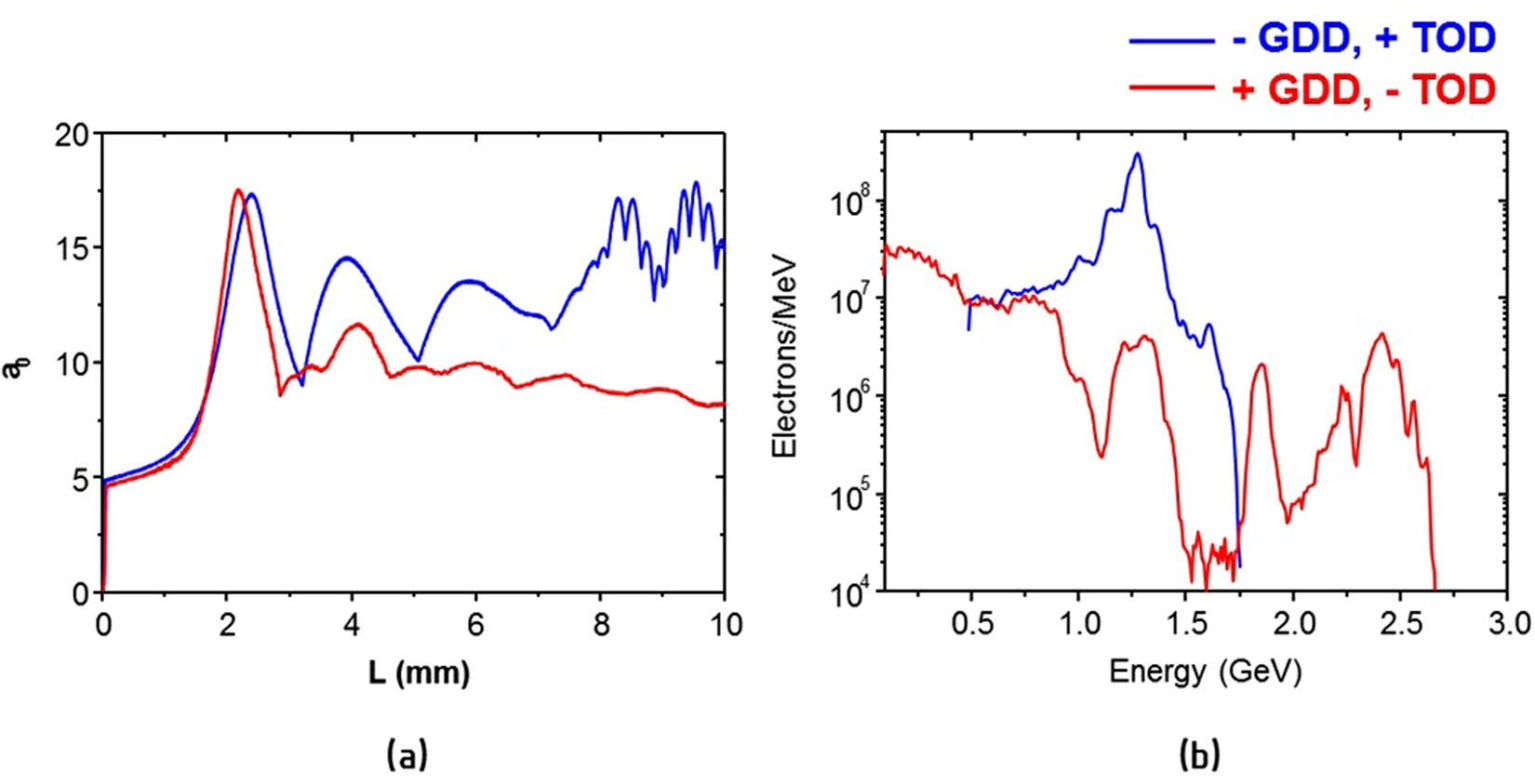
(**a**) Evolution of the normalized vector potential of a laser pulse propagating through the 10-mm long plasma medium and (**b**) electron energy spectrum after the medium obtained from quasi-cylindrical 3D PIC simulations CALDER-Circ. The blue line indicates the results obtained with the fast-rising, negatively chirped laser pulse with GDD of −400 fs^2^ and TOD of +5000 fs^3^. The red line is for the slow-rising, positively chirped laser pulse with GDD of +450 fs^2^ and TOD of −7000 fs^3^. The pulse duration in FWHM was 59 fs for both cases.

In order to interpret the experimental results, we performed the CALDER-Circ. simulations^36^ including GDD and TOD effects. In the simulations, the spectral intensity and phase taken from the experiments were used. Figure 5 shows the evolution of the normalized vector potential of a laser pulse and the final electron energy spectrum for the slow-rising positively chirped pulse and the fast-rising negatively chirped pulse. The laser propagation in the simulation showed significantly different behavior after 2.5-mm propagation. In the case of the fast-rising negatively chirped pulse, the laser field experienced strong modulation during the propagation. On the contrary, the propagation of the slow-rising positively chirped pulse is very smooth up to the end of the medium after the strong self-focusing region of the first 3-mm propagation, which can provide uniform acceleration field over a long distance. The highest electron energy peak from the slow-rising positively chirped pulses was 2.4 GeV - an enhancement of a factor of two, compared to the fast-rising negatively chirped pulse, as shown in Fig. 5(b). Therefore, the simulation results show that the propagation of a laser pulse through a plasma medium and the subsequent electron acceleration are very sensitive to the spectral phase of a driving laser pulse, which confirms that the waveform control technique for LWFA is an essential method to obtain stable multi-GeV electron beams using PW laser pulses.

## Discussion

The effects of laser chirp on LWFA have been studied using a couple of different approaches. By detuning the grating distance in a pulse compressor, as reported by Leemans *et al*. in 2002, the LWFA process can be investigated. In the study at LBL, there was a principal limitation because the detuning of grating distance accompanies the change of higher-order dispersion, inducing an asymmetric pulse profile at the same time. The study, thus, interpreted the experimental results as the effect of skewness of the laser pulse, because the ponderomotive force, the driving force of LWFA, is proportional to the first derivative of a laser intensity profile. Therefore, the all-optical programmable control of laser pulse is an essential technology to investigate systematically the laser chirp and skewness effects on LWFA.

Theoretical studies based on analytic theories and PIC simulations showed that the frequency chirp of driving laser pulses without the skewness can significantly affect LWFA, while the effect of laser chirp has not been systematically investigated in experiment. Kalmykov *et al*. reported that the dark current in LWFA can be suppressed by negatively chirped laser pulses^31, 32^. Pathak *et al*. showed that the effect of laser chirp in the linear and nonlinear regimes are different^30^. These reports showed that a positively chirped pulse in a plasma with electron density much higher than the injection threshold can generate a stronger nonlinear plasma wave than a negatively chirped pulse, resulting in continuous injection and degradation of electron energy.

We present here the experimental and theoretical results showing that a positively chirped pulse in a low density plasma near the injection threshold can enhance the electron energy and stability by providing clear electron injection and stable plasma wave structure. Since a positively chirped pulse can create stronger and stable plasma waves in the low density plasma, the acceleration gradient by a positively chirped pulse is larger than a negatively chirped or a chirp-free laser pulse. The stability of the electron beam can be also improved by the positively chirped pulse because the effects of laser fluctuations can be reduced by a strong plasma wave formed by the positively chirped pulse. Consequently, the laser chirp condition near the injection threshold drastically affected the quality of the accelerated electron beam; especially positively chirped laser pulse enhanced the energy and stability of electron beam by forming a strong plasma wave.

## Conclusion

We demonstrated the enhancement of the LWFA by systematic manipulation of spectral phase components, GDD and TOD, of PW laser pulses and achieved the generation of stable 2-GeV electron beams. The results showed that the electron energy could be drastically enhanced with positively chirped laser pulses and increased further by adding a negative quadratic chirp. In addition, the stability of the electron beams at the optimized spectral phase condition was significantly improved in energy, total charge, divergence and beam pointing. The demonstrated waveform control method for LWFA is robust, simple and easy to implement in an ultrahigh intensity laser chain, and it promises to be a powerful tool to provide stable multi-GeV electron beams for compact synchrotron x-rays^37^ and Compton gamma-rays^38, 39^ that can make innovation in nanotechnology and nuclear science.

## Methods

### PW laser system and spectral phase control

The laser-wakefield-electron-acceleration experiment has been performed using a petawatt Ti:sapphire laser^40^ operating at a 0.1-Hz repetition rate. The laser system based on a chirped-pulse amplification technique is composed of a 1-kHz multipass-amplifier front-end, a grating stretcher, a pre-amplifier, two power amplifiers, three-pass booster amplifier, and a grating compressor, delivering pulses with 30-fs duration and 30-J energy after the pulse compressor. The PW laser pulse has an amplified spontaneous emission level of 10^−8^ at 100 ps before the main pulse. A deformable mirror was installed before the compressor in order to pre-compensate for the wave-front aberration measured near the target position.

The spectral phase of PW laser pulses was controlled with sufficient dynamic ranges using two AOPDF systems installed after the pulse stretcher. The spectral phase was precisely measured with SRSI (Wizzler, Fastlite)^19^ and feedback to the two AOPDF (Dazzler, Fastlite)^18^ to make flat phase over the whole spectrum. Figure 1(i) shows the laser spectrum with almost flat phase over the full spectral range. Figure 1(ii) shows the measured temporal profile of the laser pulse (black line) and the transform-limited pulse shape (red line) for the laser spectrum. The very close matching between the two profiles indicates that the laser pulse contains no spectral chirp. After obtaining the flat spectral phase condition, we controlled the GDD and TOD of the laser pulse with the two AOPDF’s in order to synthesize the temporal shape of the laser pulse.

### Experimental setup

The LWFA experiments were carried out using the experimental setup shown in Fig. 1. The PW laser can deliver pulses with the maximum energy of 30J and the pulse duration of 28 fs. As shown in Fig. 1, a laser pulse with 26-J energy was focused with a spherical mirror having a focal length of 6 m onto a gas cell (SourceLab, SL-ALC-HI^41^) and the focused beam size was 30 μm (FWHM). The gas cell has a uniform medium density along the laser axis and the medium can vary in length from 0 to 20 mm while maintaining medium density. The maximum intensity at the focal plane was 5.5 × 10^19^ W/cm^2^, corresponding to the normalized vector potential, a_0_, of 5, for a chirp-free pulse. The electron density in the 10-mm He cell was 1.4 × 10^18^ electron/cm^3^ with a uniform distribution in the cell with density tails of 2 mm before the entrance and after the exit of the gas cell. The Thomson scattering signal from the plasma channel was recorded with a 12-bit visible CCD installed perpendicular to the laser polarization. We installed 3 phosphor screens (Kodak, Lanex) to monitor accelerated electron beams; one was set in front of the dipole magnet to capture an electron beam profile with a 12-bit CCD and two were installed behind the dipole magnet for recording energy spectra with intensified CCD’s. These two Lanex films were separated by a half-meter and the electron energy was unambiguously determined by measuring the position of electron beams on the two Lanex screens. The dipole magnet has the magnet field strength of 1.33T and the length of 30 cm. With this setup we could record the beam profile and the energy spectrum of an electron beam at each shot of temporally structured PW laser pulses.

### Simulations

We performed quasi-3D simulations using the Calder-Circ. PIC code^27^. In the simulations, we used the actual spectral intensity profile, GDD, and TOD values taken from the experiments, while the laser pulse had a Gaussian profile in the transverse direction with a flat wavefront. This fully electromagnetic PIC code solves Maxwell equations in the cylindrical coordinates (r,z), in addition to Fourier modes in the poloidal direction. Simulations were performed using two Fourier modes (m = 0 and m = 1) and with a cell size of 0.2 × 2 k_0_^−1^ in the longitudinal and radial directions, respectively. The laser intensity was 5.5 × 10^19^ W/cm^2^ for the chirp-free pulse. The plasma density was set to 2 × 10^18^ cm^−3^. Simulations were performed for two cases - positive chirp with negative TOD and negative chirp with positive TOD. The positively chirped pulse presents a slow-rising at the leading edge of the pulse, whereas the latter case presents a fast-rising profile.

## Electronic supplementary material

Supplementary informations
