# Supplementary material for: Stable multi-GeV electron accelerator driven by waveform-controlled PW laser pulses
Source: Sci Rep. 2017 Aug 31;7:10203. doi: 10.1038/s41598-017-09267-1 (PMC5579019; doi:10.1038/s41598-017-09267-1)
Supplement: Supplementary file 1 — Supplementary informations [file 41598_2017_9267_MOESM1_ESM.pdf]

Supplementary on

Stable multi-GeV electron accelerator
driven by waveform-controlled PW laser pulses

Hyung Taek Kim[1,2], V. B. Pathak[1], Ki Hong Pae[1,2], A. Lifschitz[3], F. Sylla[4], Jung Hun Shin[1], C. Hojbota[1,7], Seong Ku Lee[1,2], Jae Hee Sung[1,2], Hwang Woon Lee[1], E. Guillaume[3], C. Thaury[3], Kazuhisa Nakajima[1], J. Vieira[5], L. O. Silva[5], V. Malka[3,6], and Chang Hee Nam[1,7**]*

[1]*Center for Relativistic Laser Science, Institute for Basic Science (IBS), Gwangju 61005, Korea*

[2]*Advanced Photonics Research Institute, GIST, Gwangju 61005, Korea*

[3]*Laboratoire d'Optique Appliquée (LOA), ENSTA ParisTech, CNRS UMR7639, École Polytechnique, Université Paris-Saclay, 828 Boulevard des Maréchaux, 91762 Palaiseau, France*

[4]*SourceLAB SAS, 86 rue de Paris, 91400 Orsay, France.*

[5]*GoLP/Instituto de Plasmas e Fusão Nuclear, Instituto Superior Técnico, Universidade de Lisboa, Lisbon, Portugal*

[6]*Weizmann Institue for Science, P.O. Box 26 Rehovot, 76100, Israel*

[7]*Dept. of Physics and Photon Science, GIST, Gwangju 61005, Korea*

1. Particle-in-cell simulation for different laser chirp

We have performed a set of 2D PIC simulations using OSIRIS [1] in order to figure out the role of linear frequency chirp in LWFA process. We performed PIC simulations with various conditions with OSIRIS 2D and Calder-Cir. for the physical interpretations of experimental observations. We performed different sets of calculations to efficiently use the computational resources. For the 2D PIC simulation with OSIRIS, simulation box (x-z) of dimensions $35\times 48(c/\omega_p)^2$, moving with the speed of light along the z direction, is constructed and divided into 480×6700 cells with 4×4particles per cell. The plasma density increases linearly from 0 to 1×10^{18} electrons/cm^3 at the first 2-mm of the medium, and afterwards it is considered constant for the rest of the medium. A linearly polarized laser pulse with peak normalized vector potential $a_0 = 6$, central frequency $\omega_0 = 40\omega_p$, pulse length $L_{FWHM} = 3.4c/\omega_p$ (τ_0=50 fs), and transverse

spot size $W_0 = 5.6c/\omega_p$ (30 μm) is initialized in the simulation box with linear chirp coefficient $\pm 1.0\omega_p^2$. The transverse profile of the laser pulse closely resembles the experimental laser pulse with side-wings. The peak of the side-wing is located at 45 μm away from the laser axis with Gaussian transverse profile of 10-μm width and the normalized vector potential is 10% of the main peak a_0.

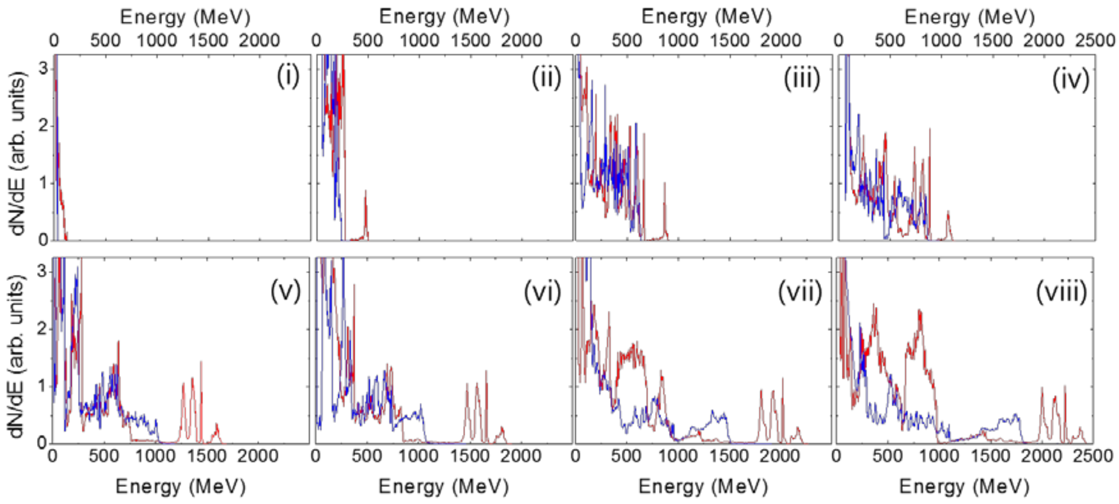

Figure S1. Electron energy spectra for positively (red line) and negatively (blue line) chirped pulses at z= 2.56 (i), 3.45 (ii), 4.49 (iii), 5.2 (iv), 6.7 (v), 7.4 (vi), 8.57 (vii), and 10 mm (viii), where z is the propagation distance of the laser pulse.

The electron energy spectra, taken at several longitudinal positions from the PIC simulations performed with positively and negatively chirped pulses, are shown in Fig. S1. For the positive chirp case, a mono-energetic electron bunch over 500 MeV is observed at z=3.6 mm, while continuous electron energy spectrum below 500 MeV is observed for the negative chirp case. The electron energy for the positive chirp increases rapidly and reaches 1.8 GeV at z=7.4 mm, while the electron energy for the negative chirp saturates to 1 GeV. As the laser further propagates, the electron energy for the positive chirp increases slowly up to 2.4 GeV. On the other hand, for the negatively chirp pulse the electron energy only reaches to 1.7 GeV at the end of the medium. Overall, the positively chirped pulse provides well defined electron injection and higher acceleration gradient in the early stage of acceleration than the negatively chirp pulse. These

simulation results qualitatively show that the positively chirped laser pulses can provide higher acceleration field within 10 mm acceleration length.

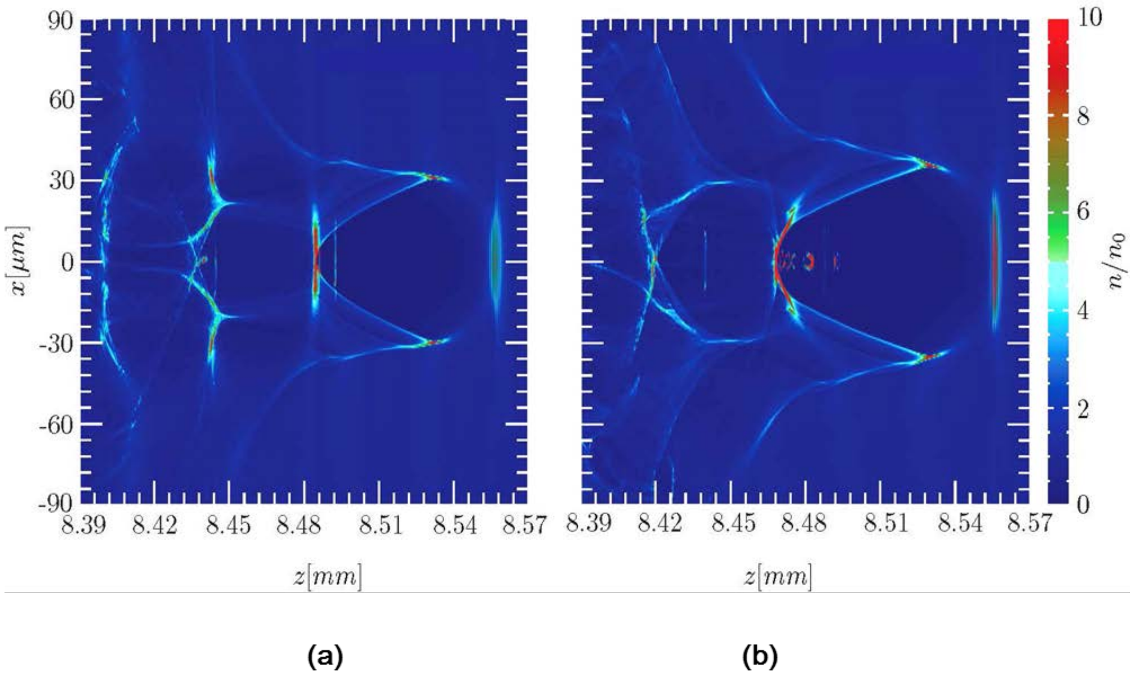

Figure S2. Electron density distribution for the laser position at z=8.57 mm for (a) the negatively chirped and (b) the positively chirped 50-fs laser pulses.

We look into the electron density distribution for the case of the laser position at 8.57 mm in order to find out the effect of frequency chirp on the bubble structure. Figure S2 shows the electron density distribution for the negative and the positive laser chirps. With a weak side-wing in the beam profile, the wall of the plasma bubble becomes slightly elongated, as compared to the case without a wing structure. Due to the higher ponderomotive potential in the leading edge of the positively chirped pulse a larger and stronger plasma bubble is created in the positive chirp case than in the negative chirp case, which makes well-confined electron beams with higher energy, as shown in Fig. S2 (b). With the propagation through the plasma medium, the higher rate of localized etching at the leading edge of the positively chirped pulse can contribute to the formation of a stronger and more stable bubble, as compared to the case of a negatively chirped pulse. In the case of the negative chirp, the disturbance in the side-wing is quite significant and the electron bunches are spread along the transverse direction due to the dispersed bubble wall. In addition, the etching

of the positively chirped pulse is stronger than that of the negatively chirped pulse [2], which enhances the effect of the strong ponderomotive push by the positively chirped pulse as the laser pulse propagates through the plasma medium. Consequently, the positively chirped pulse generates strong ponderomotive potential at the pulse front and suitable plasma bubble structure for electron acceleration, providing the conditions to achieve electron beams with high energy.

2. Electron beam profiles for difference laser chirps

Figure S3 shows the typical electron beam profile experimentally measured at the LANEX 1 for three different laser chirps: (i) positively chirped 40-fs, (ii) chirp-free 28-fs, and (iii) negatively chirped 40-fs laser pulses. In the profile, we could not observe significant change of total charge for different laser chirp. The positively chirped pulse roughly 20 % higher beam charge than the others. Since the electron density of the plasma medium is very near to the injection threshold, the serious continuous injection by the positively chirped pulse can be inhibited by the low electron density. In the simulations with difference GDDs, we also could not observe noticeable differences of injected charge into the plasma bubble at this low electron density. In addition, the shape of the electron beams was not sensitive to the laser chirps, while the electron beam for positive chirp case having slightly smaller beam divergence along the laser polarization axis.

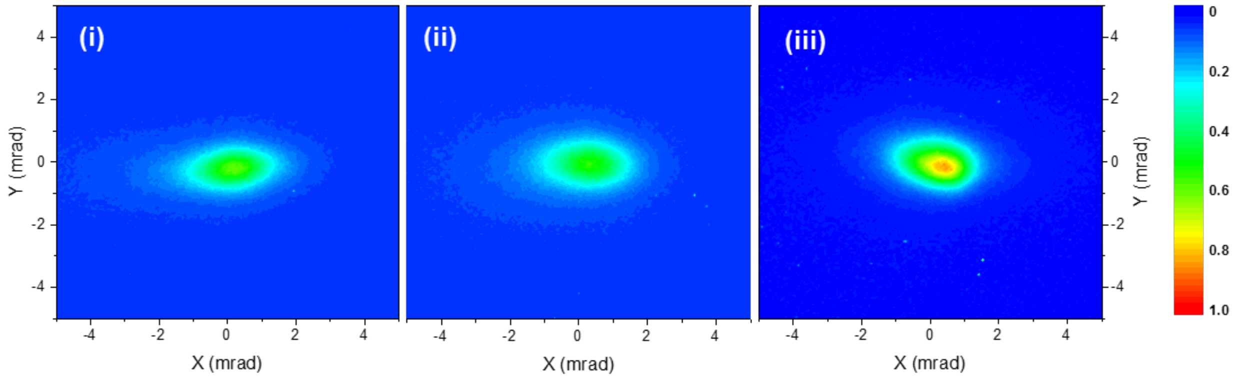

Figure S3. Typical electron beam pattern measured by LANEX screen in front of dipole magnet for the cases of (i) positively chirped 40-fs, (ii) chirp-free 28-fs, and (iii) negatively chirped 40-fs laser pulses. The laser field was polarized along the horizontal axis.

[1] Fonseca, R. A. *et al.* OSIRIS: A Three-Dimensional, Fully Relativistic Particle in Cell Code for Modeling Plasma Based Accelerators Lecture Notes in Computer Science **2331**, 342-351 (2002);

[2] Pathak, V. B., Vieira, J., Fonseca, R. A. & Silva, L. O. Effect of the frequency chirp on laser wakefield acceleration. *New J. Phys.* **14,** 023057 (2012).
